# Immunosuppressive therapy for aplastic anemia: a single-center experience from western India

**DOI:** 10.1007/s00277-018-3487-2

**Published:** 2018-09-01

**Authors:** Sandip Shah, Preetam Jain, Kamlesh Shah, Kinnari Patel, Sonia Parikh, Apurva Patel, Harsha Panchal, Asha Anand

**Affiliations:** Civil Hospital Campus, Gujarat Cancer & Research Institute, Asarwa, Ahmedabad, Gujarat 380 016 India

**Keywords:** Immunosuppressive therapy, Aplastic anemia, Antilymphocyte serum, Antithymocyte globulin

## Abstract

Immunosuppressive therapy (IST) with antithymocyte globulin (ATG) and cyclosporine A (CsA) is the first-line therapy for acquired aplastic anemia (AA) in those not suitable for bone marrow transplant. Horse ATG (hATG) is preferred for this purpose, but its use is often impeded by shortages and costs. Being a rare disease, there is limited data on this therapy. This study aimed to evaluate this therapy in a large cohort of AA patients from western India. We retrospectively analyzed AA patients who received an indigenous preparation of hATG along with CsA as first-line treatment, between 2012 and 2015, at our center and evaluated the response, survival, and occurrence of adverse events. The response was further assessed separately for adults and children. During the period, 91 AA patients (4 non-severe, 57 severe and 30 very severe) were treated with IST. At 2 years, 23.5% adults and 39.1% children showed complete response and an overall of 68.1% cases became transfusion independent. More than half of the patients developed febrile neutropenia while roughly one sixth of the patients developed gum hypertrophy and/or hypertension. Two patients had clonal evolution. Mortality rate was calculated to be 31%; most common causes of death were infection and intracranial hemorrhage. The results of the study substantiate the effectiveness of IST in AA, using an inexpensive indigenous preparation of hATG along with CsA.

## Introduction

Acquired aplastic anemia (AA) is a rare disease and, in its severe form, potentially fatal. It is characterized by hypocellular bone marrow and pancytopenia. The underlying pathophysiology is believed to be immune-mediated T cell destruction of hematopoietic stem cells [1, 2]. A biphasic age distribution with peaks at 15–30 years and ≥ 60 years is seen [3, 4]. The incidence is believed to be 2–3 times higher in Asia compared to the West, where the estimated incidence rate of AA is 2 per million people per year [1, 2, 4]. Allogeneic hematopoietic stem cell transplantation (HSCT) from the bone marrow of a human leucocyte antigen (HLA)-matched sibling donor (MSD) is regarded as the initial treatment of choice for newly diagnosed patients with severe AA (SAA). For patients where no MSD is available, which can be expected for 70% of patients with SAA, immunosuppressive therapy (IST) is indicated as first-line therapy [2, 3]. Both modalities have been found to result in similar survival rates (> 80%) [5]. A recent Cochrane systematic review reported that survival ranged from 47 to 84% with MSD-HSCT and from 45 to 87% with IST [1].

Since the early 1990s, antithymocyte globulin (ATG) along with cyclosporine A (CsA) has been considered the standard IST for AA patients, with an expected 50–60% probability of response and 60% overall survival at 1 year [6]. Children show higher rates of recovery and survival [2, 3]. The beneficial effects of this regimen are believed to be due to the ability of the polyclonal antibodies in ATG to recognize a variety of human lymphocyte cell surface antigens, reduce lymphocyte numbers, and induce an immunosuppressive effect, which is further helped by the specific inhibitory effects of CsA on T lymphocyte function. Multiple studies have reported better outcomes with the use of horse ATG (hATG) compared to rabbit ATG (rATG) [7–9]. Some studies have, however, reported similar efficacy with the two preparations when the patients are followed up beyond 6 months, indicating a longer response time required with rATG than with hATG [10, 11]. Two recent meta-analyses have also reported mixed findings, with one analysis reporting significantly higher response rate with hATG compared to rATG [12] and the other reporting superior response of hATG up to 6 months but similar early mortality and evolution with both preparations [13]. However, due to the lack of any study showing superiority of rATG, the general agreement is to prefer hATG for IST in AA and use rabbit ATG only when hATG is not available or in resistant cases [9, 14].

Owing to the rarity of the condition, there are few studies published with a limited number of patients and fewer studies from the developing world. With an aim to generate more data on the effectiveness of this therapy, we have conducted a retrospective study in a large cohort of AA patients treated with IST at our institute, located in western India.

## Materials and methods

### Site

The study was conducted at a state-owned cancer research institute, located at Ahmedabad, an important economic and industrial hub in west India with a population of > 6 million. Approval of the institutional ethics committee was obtained prior to the conduct of the study.

### Study design

This is a retrospective case record analysis of patients diagnosed with AA and treated at the institute with IST. The clinical course of the patients has been evaluated with regard to response rates to the treatment and survival over a maximum follow-up period of 24 months.

### Population

Medical records of all patients with AA, admitted between January 2012 and December 2015, were screened for inclusion in the study. Only cases with newly diagnosed AA and treated with IST were included in the study. Pregnant females, patients who tested positive for HIV and those diagnosed with Fanconi’s anemia were excluded from the study. The study population was further divided into different severity strata—non-severe AA (NSAA), severe AA (SAA), and very severe AA (VSAA), as per the criteria given by Camitta [15] and Bacigalupo [16], which is referred to by the British Committee for Standards in Haematology (2009) guidelines for the diagnosis and management of aplastic anemia.

### Intervention under study

Thymogam® (manufactured by Bharat Serums and Vaccines Ltd.), an indigenous preparation of hATG, is available in our institute under a state-sponsored scheme and was used for IST during the study period, too. Most patients received hATG, administered at a dose of 40 mg/kg/day for 4 days through a central line over 4–6 h. There were 3 patients who received ATG at a dose of 20 mg/kg/day for 8 days. Hydrocortisone and pheniramine were administered as premedication before each dose of ATG. Intravenous methylprednisolone was used prophylactically for the prevention of serum sickness. CsA was initiated at a dose of 10 mg/kg along with hATG and continued until the patient had become transfusion independent or for a minimum of 1 year with periodic monitoring of blood levels to maintain the drug level between 200 and 500 ng/ml. Other supportive treatments included red cell or platelet transfusions, hemopoietic growth factors, and prophylaxis or treatment of infections with antibiotics.

### Data

Basic patient material was identified from the medical records of the hospital. Data were obtained on a pre-designed case record form (CRF) from the archived patients’ files. Data on patient demographics, blood counts, marrow picture, duration of IST therapy, complications, and concomitant medications were extracted from the archived patients’ files.

### Outcomes

The outcomes of interest were hematological response rate (partial and complete), mortality, incidence of complications/adverse effects, and their clinical outcomes. The hematological response rate was recorded as complete response (CR) or partial response (PR) as per the criteria given in British Committee for Standards in Haematology (2009) guidelines for the diagnosis and management of aplastic anemia [3]. The follow-up time-points for assessment of response were baseline and 3 months, 6 months, 9 months, 12 months, 18 months, and 24 months from the initiation of IST. With an intent-to-treat approach, patients who did not follow-up were presumed to have not responded and died. The outcomes were analyzed for the whole study population as well as separately for the different age groups.

### Statistical analysis

Data was collated in a Microsoft® Excel® spreadsheet and statistical analysis was performed using SAS® software version 9.4. The demographic and baseline characteristics have been summarized using descriptive statistics [number of patients (n), mean, standard deviation (SD), median]. Response to IST, mortality, and adverse events have been summarized using frequency counts (n) and percentages (%).

## Results

### Patient characteristics

Ninety-one patients were included in the study. Majority (62.6%) of the patients belonged to the SAA, while 33% belonged to VSAA group and 4.4% belonged to NSAA group. There were twice as many males (60) as there were females (31). Sixty-eight of the patients were adults, while 23 were children. The median age of the studied adult patients was 29 years, while the median age of children was 10. Summary demographic and baseline data is provided in Table 1.

**Table 1:** Demographic and baseline characteristics

Parameter		Value	
Total number of subjects		91	
Severity of AA [n (%)]	VSAA	30 (33)	
	SAA	57 (62.6)	
	NSAA	4 (4.4)	
		Adults (*n* = 68)	Children (*n* = 23)
Age [years (median)]		29	10
Sex (male:female)		46:22	14:9
Hemoglobin [gm/dl (Mean ± SD)]		7 ± 2.1	7.9 ± 1.9
ANC [per μL (Mean ± SD)]		567 ± 468	614 ± 523
Platelets [per μL (Mean ± SD)]		12,191 ± 11,025	15,173 ± 13,721

*VSAA* very severe aplastic anemia, *SAA* severe aplastic anemia, *NSAA* non-severe aplastic anemia, *ANC* absolute neutrophil count

### Response rates

Summary of the number of subjects showing response to IST at different time-points is provided in Table 2. All remissions were confirmed by 2 blood counts at least 4 weeks apart. At the final follow-up, 62 (68.1%) patients were transfusion independent.

**Table 2:** Number of patients showing response at different time-points

Time-point	Overall (*n* = 91)		Adults (*n* = 68)		Children (*n* = 23)	
	CR	PR	CR	PR	CR	PR
3 months	2 (2.2%)	36 (39.6%)	1 (1.5%)	24 (35.3%)	1 (4.3%)	12 (52.2%)
6 months	8 (8.8%)	36 (39.6%)	6 (8.8%)	24 (35.3%)	2 (8.7%)	12 (52.2%)
12 months	17 (18.7%)	34 (37.4%)	12 (17.6%)	23 (33.8%)	5 (21.7%)	11 (47.8%)
24 months	25 (27.5%)	37 (40.7%)	16 (23.5%)	30 (44.1%)	9 (39.1%)	7 (30.4%)

*CR* complete response, *PR* partial response

### Mortality

Twenty-eight (31%) patients died in the follow-up period. Mortality rates and their causes have been summarized in Table 3.

**Table 3:** Mortality over the follow-up period

Groups	Mortality [*n* (%)]
Overall (*n* = 91)	28 (30.8)
Early (< 3 months)	11 (12.1)
Late (≥ 3 months)	17 (18.7)
Adults (*n* = 68)	22 (32.4)
Children (*N* = 23)	6 (26.1)
Cause of death	*n* (%)*
Infection	11 (39.3)
Pneumonia	7 (25)
Sepsis	4 (14.3)
Intracranial hemorrhage	8 (28.6)
Unknown/confirmed on telephone	7 (25)
Renal failure	1 (3.6)
Acute myeloid Leukemia	1 (3.6)

*Denominator is the total number of deaths

### Adverse events

Febrile neutropenia was the most common (affecting 57.1% patients) adverse event reported, followed by gum hypertrophy (15.4%), hypertension (14.3%), pneumonia, intra-cranial hemorrhage, and elevated creatinine level. None of the patients developed serum sickness. Two patients had clonal evolution—1 developed AML and died at 6 months after IST, the other developed acute lymphoblastic leukemia (ALL) at 7 months after IST after being in CR for 4 months and was put on Berlin-Frankfurt-Münster (BFM) 90 chemotherapy regimen [17] (which involves stratification of treatment intensity based on resectability, lactate dehydrogenase level and stage). Summary data of adverse events is provided in Table 4.

**Table 4:** Adverse events following IST in survivors

Event	*n* (%)
Febrile neutropenia	52 (57.1)
Gum hypertrophy	14 (15.4)
Hypertension	13 (14.3)
Pneumonia	5 (5.5)
Intracranial hemorrhage	5 (5.5)
Raised creatinine/renal Failure	2 (2.2)
Serum sickness	0 (0)

## Discussion

There is a paucity of literature pertaining to responses with ATG and cyclosporine in AA from India, with most of the published studies including around 20–30 patients. This is a retrospective case record analysis of a large cohort of 91 AA patients from a single center examining the role of IST, comprising of hATG and CsA. Majority (95.6%) of the patients included had severe disease, with one third of the patients having very severe disease. The study demonstrates that approximately 70% of patients can have long-term survival with IST, using an indigenous hATG preparation.

Many studies conducted across the world have reported response rates ranging from 50 to 78% to hATG-based IST in AA, over a period of 3 to 12 months [18–23]. Previous studies in India have reported varied results ranging from 33.3 to 87.9% response rates [24–29]. In our study, an overall response in adult patients, at 6 months, was seen in 44.1%, and at 24 months, as many as 67.6% of the patients had responded. Pediatric patients appeared to respond faster as more than half the patients showed response at 3 months itself; the same feat was achieved in the adult patients at 12 months. The overall response in pediatric population was also slightly higher at 69.6%, at the end of 24 months. The results in children are much better than those reported in earlier Indian studies by Sharma et al. [24], Chandra et al. [25], and Gupta et al. [26]. Table 5 shows the comparison of responses to IST in different studies (Indian studies provided separately).

**Table 5:** Summary of studies on IST using hATG and CsA in AA

Study	IST used	Design	*N* (total)	Age group (yrs)	Responders with hATG + CsA
Scheinberg_2009 [18]	hATG^A^ + CsA	Prospective	77 (42*)	4–78	At 3 months: 50% At 6 months: 62%
Teramura_2007 [19]	hATG^C^ + CsA	Prospective	101 (50*)	18–75	At 3 months: 51% At 6 months: 57% At 12 months: 76%
Rosenfeld_2003 [21]	hATG^A^ + CsA	Prospective	122	Not specified	At 3 months: 60% At 6 months: 61% At 12 months: 58%
Frickhofen_2003 [22]	hATG^D^ + CsA	Prospective	84	Not specified	At 4 months: 70%
Marsh_1999 [23]	hATG^C^ + CsA	Prospective	115 (54*)	1–67	At 6 months: 74%
Rosenfeld_1995 [20]	hATG^A^ + CsA	Prospective	55	4–79	At 3 months: 67% At 6 months: 71% At 12 months: 78%
Indian studies					
Patel_2015 [29]	hATG + CsA	Retrospective	18	7–58	At 6 months: 43.8% At 12 months: 43.8% At 18 months: 50%
Gupta_2012 [26]	hATG^B^ + CsA	Retrospective	30	4–14	At 6 months: 33.3%
Sharma_2012 [24]	hATG^A/B/C^ + CsA	Retrospective	35	5–12	At 12 months: 50%
Chandra_2008 [25]	hATG^A/B/C^ + CsA	Prospective	23	6–12	At 6 months: 40%
Nair_2012 [27]	hATG^A^ + CsA	Prospective	33	7–18	At 6 months: 87.9%
Agarwal_2015 [28]	hATG^B^ + CsA	Prospective	30	9–58	At 3 months: 40% At 6 months: 50%
Current study	hATG^B^ + CsA	Retrospective	91	2–67	At 3 months: 41.8% At 6 months: 48.4% At 12 months: 56% At 24 months: 68.1%

*On ATG + CsA therapy, ^A^atgam, ^B^thymogam, ^C^lymphoglobuline, ^D^lymphoglobulin

The most common adverse effects observed in this study—febrile neutropenia, gum hypertrophy, and hypertension—are in line with those reported in other such studies [26, 29]. Allergic reactions, like serum sickness, which are anticipated and reported in a substantial proportion of ATG recipients [19, 30] are effectively prevented by prophylactic administration of anti-allergic medications, as seen in this study.

Although hATG and CsA combination is accepted as the standard immunosuppressive regimen for patients with SAA and for those not suitable for HSCT, the main problem, that hATG is expensive and its availability is riddled with uncertainties, remains. Many countries, in Europe and Asia, are forced to use rATG due to unavailability of hATG [3, 31, 32]. In India, only two preparations of hATG are available—Atgam (Pfizer) and Thymogam (Bharat Serums and Vaccines), with the former being around three times as expensive as the latter. In a country like India, which is ranked outside the first 150 countries based on the Gross domestic product (GDP—per capita—purchasing power parity) [33], cost constraints prevent the use of IST in a large number of eligible patients [26, 29]. In this context, the availability of an indigenous preparation of hATG (Thymogam) in India, with efficacy and safety comparable to that reported globally, and its distribution through a state-sponsored scheme, as in our institute, is a boon for the less-affording patients.

This was a retrospective case-record analysis and hence there was no scope to control any known or unknown factor that could influence the response rates, including concomitant medications, duration since diagnosis, and treatment practices of the center.

In summary, the efficacy and safety results obtained in the present study are comparable to those reported in global studies and serve to substantiate the effectiveness of IST in AA, using an inexpensive indigenous preparation of hATG along with CsA.
